# Co-infection with *Streptococcus anginosus* and *Mycobacterium tuberculosis* in an immunocompetent pediatric patient. A case report

**DOI:** 10.1186/s12890-019-1044-y

**Published:** 2020-01-08

**Authors:** Napoleon González Saldaña, José Iván Castillo Bejarano, Marte Hernández Porras, Eduardo Arias de la Garza, Sofia Fortes Gutiérrez, Jose Luis Copado Gutiérrez, Hugo Juarez Olguin

**Affiliations:** 1 Department of Infectious Diseases, National Institute of Pediatrics (NIP), Imán Avenue No. 1, 3rd floor, Cuicuilco District, 04530 Mexico City, CP Mexico; 2 Laboratory of Pharmacology, NIP and Dept of Pharmacology Faculty of Medicine, Universidad Nacional Autónoma de, Mexico City, Mexico; 3 Laboratorio de Farmacología, Instituto Nacional de Pediatría, Avenida Imán N° 1, 3rd piso Colonia Cuicuilco CP, 04530 Mexico City, Mexico

**Keywords:** Tuberculosis, *Streptococcus anginosus*, *Mycobacterium tuberculosis*, Pediatrics, thoracic abscess

## Abstract

**Background:**

Simultaneous infection in tuberculosis (TB) is rare. The mixed infection between *Streptococcus anginosus* group (SAG) and *M. tuberculosis* (MTB) has not been reported in children. The aim of this report was to describe a pediatric case with a pulmonary abscess caused by the duality SAG-MTB co-infection.

**Case presentation:**

An 11-year-old boy with an acute onset of throbbing pain of two-day evolution located in the anterior chest wall. The patient reported a history of fever, cough and rhinorrhea during the last seven days. An anterior chest radiography revealed a heterogenic opacity at the lower right lobe while the lateral projection showed an obliteration at the anterior diaphragmatic insertion. Parenteral Ceftriaxone (100 mg/kg/day) and Dicloxacillin (200 mg/kg/day) was started. The abscess was subsequently drained and analyzed. After a year of follow-up, the patient remained asymptomatic.

**Conclusion:**

This case represents the first reported case of pulmonary co-infection involving MTB and SAG in an immunocompetent pediatric patient.

## Background

The simultaneous appearance of tuberculosis (TB) and bacterial infection is not common. A triple infection of acquired immunodeficiency syndrome, *Mycobacterium tuberculosis* (MTB) and pneumococcal pneumonia has been reported with the first being a crucial predisposing factor for the co-infection of MTB and other bacteria mainly pneumococcal pneumonia [1].

*Streptococcus anginosus* group (SAG) is part of the commensal flora of the oral cavity and the genitourinary tract. This group of pathogens is characterized by causing abscess-forming infections at various sites in the body [2]. The infection prevalence has been reported to be higher in adult males. However, as far as we know, the correlation between TB and SAG in children has not been previously described. It is important to underline that male patients could have a higher likelihood of SAG confections due to X-linked primary immunodeficiencies such as chronic granulomatous disease, although such very rare infections are not exclusive to the male gender.

In adults, the predominating sites of infection by SAG are the central nervous system (CNS), the respiratory tract and the intra-abdominal areas. In pediatric population, SAG infections are usually accompanied by abscesses in the head, neck and upper chest [3].

Predictors of pneumonia in children are fever and cyanosis plus two or more of the following respiratory distress signs: tachypnea, cough, nasal flaring, retractions, rales, and decreased breath sounds. For children older than five, the World Health Organization (WHO) defines tachypnea as a respiratory rate greater than 30 breaths per minute.

SAG has been widely found in the mouth, upper respiratory tract, gastrointestinal tract and vaginal cultures [4]. The role of SAG in the pathogenesis of respiratory infections has been recently described by Mukae et al., based on the result of clone library analysis [5]. Thoracic infections represented one-fifth of all SAG infections in one series [4]. Simultaneous infections with MTB and other agents is rare, typically involve *S. pneumoniae* and are most commonly reported among HIV-infected patients [1]. Clinically, the isolation of SAG co-infection with MTB has not been described in pediatric patients [6]. In this report, we describe a case of an abscess caused by the duality SAG-MTB co-infection in a pediatric immunocompetent. The unusual evolution of the TB picture in a pediatric patient makes this case report particularly noteworthy. In fact, a pulmonary abscess developed in a matter of days, and the only pathogen identified through BD Phoenix automated testing was SAG, a rather infrequent finding.

## Case presentation

An 11-year-old boy, resident of Mexico City and previously healthy, with a weight of 42 kg and a height of 1.32 m was admitted at the Emergency Department of National Institute of Pediatrics, Mexico City with an acute onset of throbbing pain of two-day evolution located in the anterior chest wall. The patient reported a history of fever, cough and rhinorrhea during the last seven days. When asked, the parents did not report constitutional symptoms such as weight loss, asthenia, adynamia, or night sweats. The child’s immunization record card was complete in accordance with the national recommendations and included BCG administered at birth. Family contact study performed by Epidemiology Unit involving the entire family (siblings, parents and grandparents) and those living nearby was negative with no history of TB, or any other important comorbidity in the family or close contacts. Extension studies at school were not carried out by the Epidemiology Unit. An electrocardiogram (ECG) performed at his arrival at the Emergency Department showed a right branch blockade, which was deemed as physiological. Chest radiography (CXR) did not reveal any significant findings and the patient was subsequently discharged. A day after his discharge, the patient was re-admitted for a workup due to chest pain, now located at his right thorax, along with dyspnea and fever of 38.6 °C.

## Diagnosis and treatment

**Fig. 1:**
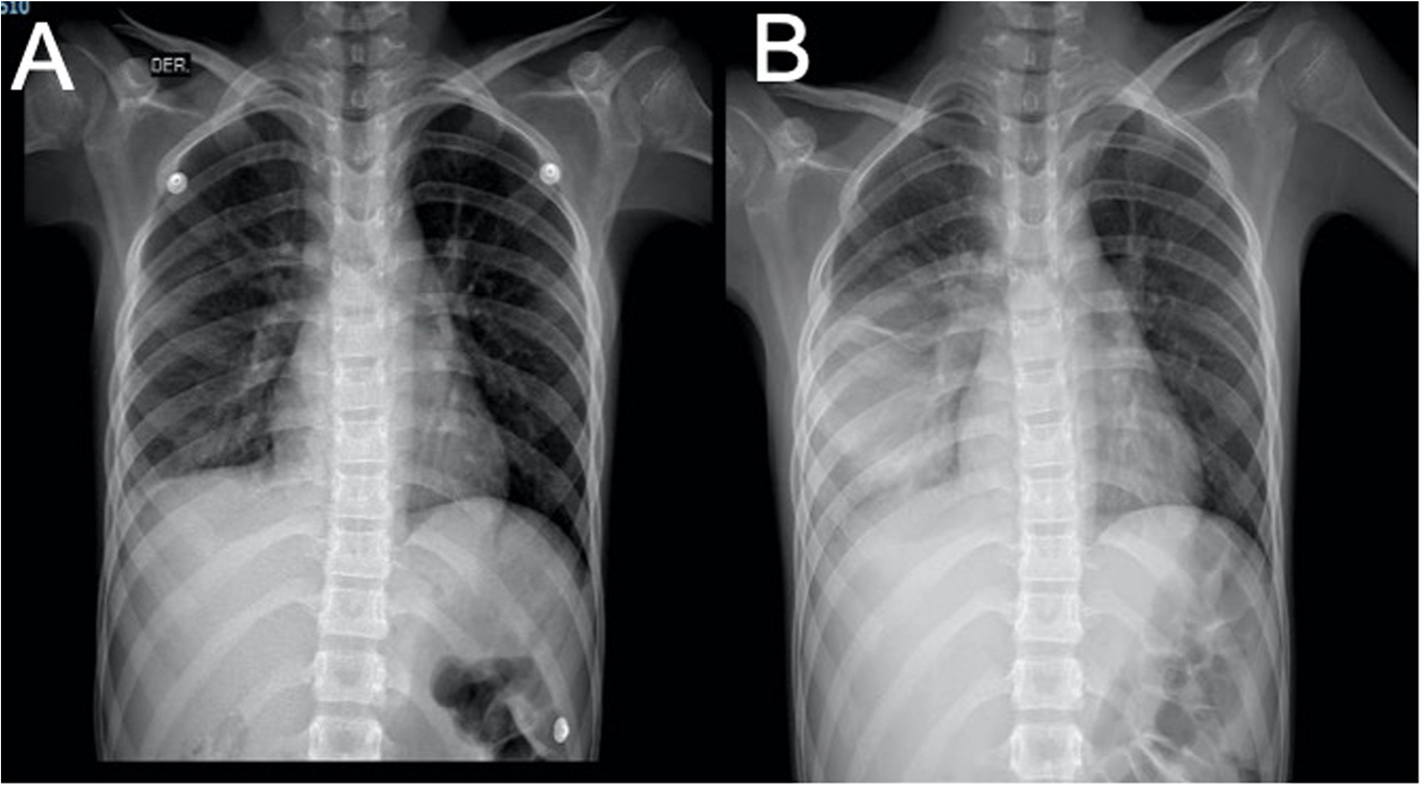
**a**) Radiography taken at admission with presence of right pleural effusion. **b**) Right basal consolidation and pleural effusion

Upon re-admission, the patient was found tachypneic with bilateral upper and lower chest retractions, without signs of hypoxia. Complete blood count (CBC) showed a slight increase (14,000/mm^3^) with a significantly high absolute neutrophil count (ANC) (13,440/mm^3^ neutrophils). An anterior chest radiography (Fig. 1a, b) was performed, revealing a heterogenic opacity at the lower right lobe, while the lateral projection showed an obliteration at the anterior diaphragmatic insertion.

A diagnosis of complicated pneumonia was made, and parenteral Ceftriaxone (100 mg/kg/day) and Dicloxacillin (200 mg/kg/day) was started. This is the treatment of choice in our hospital to cover gram-negative bacteria and staphylococci, as is common practice for the management of severe and complicated pneumonia cases. We use this scheme to cover mainly infections with *S.pneumoniae and S. aureus*, always guided by clinical and extension studies such as radiography, blood count and PCR.

**Fig. 2:**
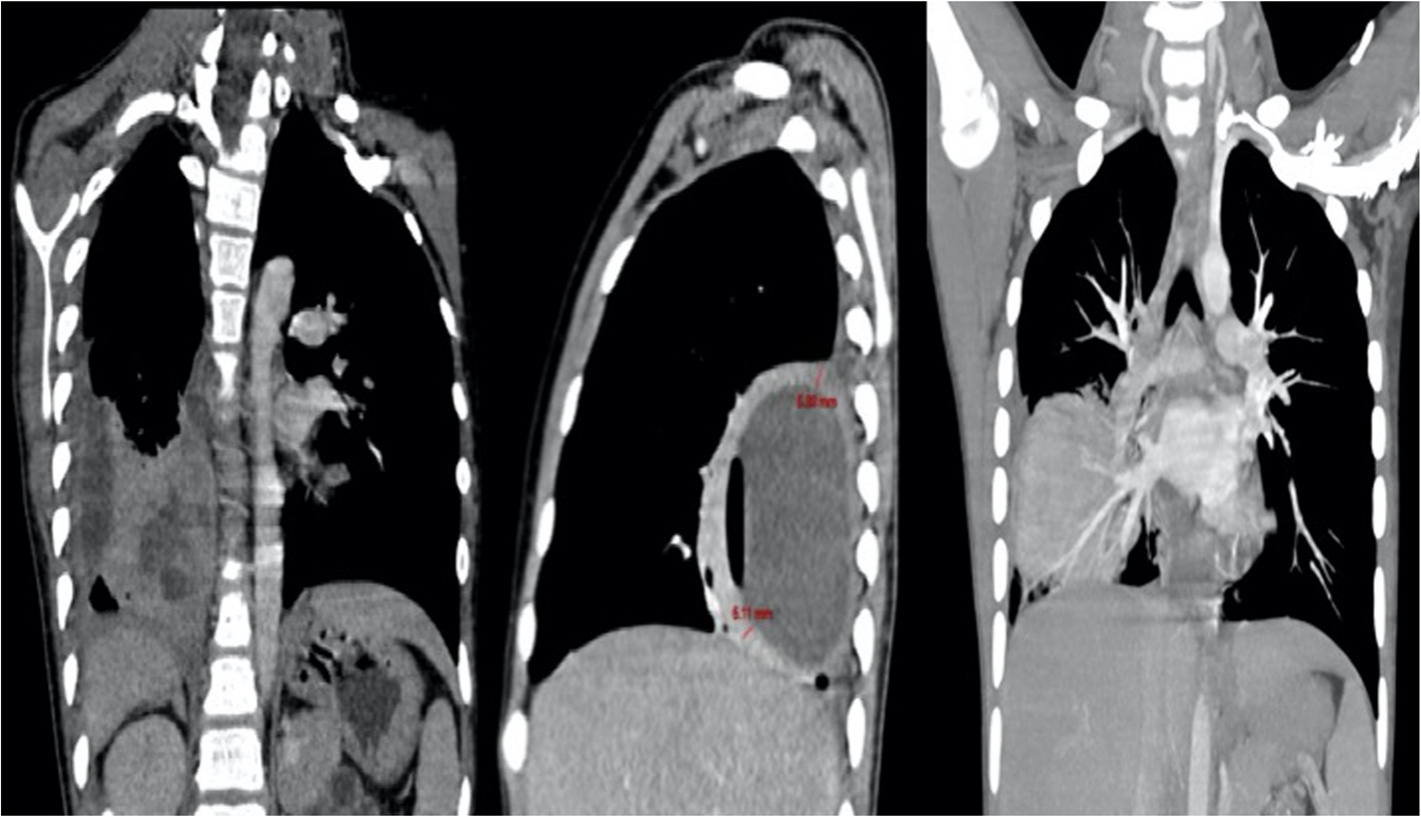
Cavitated pneumonia (abscess) in the medial, posterior and right lateral basal segments, associated with mediastinal adenopathies

However, as the patient’s improvement was below expectations, we performed a thoracic ultrasonography which revealed a septated empyema of about 260 mL along with right basal atelectasis. A chest tube was placed draining a turbid fluid with 375 cells (80% neutrophils, 8% lymphocytes, 9% monocytes and 3% eosinophils), pH 8, proteins 5.31 g/dL and glucose 24 mg/dL. Tests of LDL and Triglyceride were discarded since they are not routine tests and for this case, were considered irrelevant. Chest tube was removed after 5 days of drainage. Nonetheless, despite the drainage of the fluid and antibiotic management, the patient persisted febrile and was shifted to parenteral Vancomycin, with the presumption that *methicillin resistant Staphylococcus aureus* **(**MRSA**),** or Cephalosporin-resistant *Streptococcus pneumoniae* infection might be involved. Thirteen days after re-admission, the patient presented an episode of hemoptysis and an increase in CRP (from 8.4 mg/dL to 12.7 mg/dL). Further investigations for persistent fever were conducted, including tests for MTB as the patient came from an area with an annual TB incidence of 9,1/100,000 even if no household, or close contacts were documented. Xpert MTB/RIF was performed on a sputum sample, resulting positive for MTB complex and negative for rpoB mutations. Later drug-susceptible MTB was isolated in mycobacterial culture. A chest CT scan was performed, and an image compatible with a lung abscess of 75.7 mm × 49.18 mm was visualized (Fig. 2). After conducting a pulmonary CT scan, the TB diagnosis was confirmed. The abscess was subsequently drained and analyzed. The abscess drainage purulent secretion was subjected to a 24-h incubation in agar blood with the appreciation of the growth of small colonies of α-hemolysis. Subsequently, in a BD PhoenixTM test, SAG was identified. Analysis of the purulent secretion performed on admission in search of aerobes, anaerobes, fungi and mycobacteria, under the suspicion of a co-infection, as well as genetic testing by Xpert MTB / Rif (Cepheid Inc., CA, USA) and acid fast bacilli smear only reported SAG.

New RXs were performed after a controlled drainage, and the patient’s clinical outcome was favorable. Fever decreased in approximately 4 days after lymphatic drainage. Antituberculous therapy was started (INH/RIF/PZA/E), and Vancomycin treatment was continued for 28 days. An immunodeficiency panel depicted a normal nitro blue tetrazolium and immunoglobulin quantification, while IL-12 / gamma-IFN assay and fourth-generation HIV ELISA were negative. The patient was discharged after 37 days of stay, with an adequate response to treatment. The patient completed 2 months of intensive anti-TB treatment, and later the child was shifted to maintenance therapy with INH/RIF for 4 months. After a year of follow-up, the patient remained asymptomatic.

## Discussion

TB abscesses are infrequent in immunocompetent hosts, and spine involvement is reported in most cases [7]. Dual infections of TB and bacteria have already been described. However, this is a quite rare occurrence in immunocompetent patients, being much more common in HIV-infected subjects who are at higher risk of pneumococcal infection (i.e. the most frequent co-infecting agent) [6].

SAG bacteria are facultative anaerobic pathogens that colonize the mouth and the upper respiratory tract. Infections with SAG are commonly characterized by the formation of abscesses and pleural empyema. Okada et al., [8] reported that 54.5% of all polymicrobial infections involve SAG and that 66.7% of this population usually require early surgical treatment with a low mortality rate, and without isolation of MTB in the pus resulting from the drainage of the empyema or abscess. In another study, Noguchi et al., [4] reported 20% co-infection, including *H. influenzae*, *Bacteroides* spp. and other pathogens with a mortality rate of 6.7%.

The management approach adopted in the present case was surgical drainage of the abscess without decortication, and antibiotic therapy for a total of 35 days. Although SAG infections usually cause acute, or subacute disease in patients co-infected with TB the contribution of the pathogen may not be easily distinguishable from a clinical standpoint. It has been reported that *viridans streptococci* may inhibit MTB growth in vitro, but it remains unknown whether MTB can enhance the pathogenicity of SAG when they coexist in the same infection [9, 10]. In this case, the disease in the presence of lung TB presented with a rapid onset with pleural effusion and abscess. From the purulent drained pleural effusion and abscess, SAG was isolated in aerobic bacteria culture. The rest of the cultures were negative. This acute onset of community-acquired pneumonia has been described mainly in places with high incidence of TB and HIV [11].

Primary immunodeficiency such as Chronic Granulomatous Disease (CGD), Mendelian susceptibility to Mycobacterial diseases, Severe Combined Immunodeficiency (SCID) and secondary HIV immunity, among others, are known to be risk factors for TB disease [12]. Male gender, periodontal disease, alcohol abuse, cancer, HIV infection, and cystic fibrosis are all predisposing factors for SAG infection [13]. To our knowledge, correlation between TB and SAG has not been previously described. Our patient received 2HRZE/4HR regimen as suggest the WHO for treatment of drug-susceptible tuberculosis [14].

## Conclusion

To our knowledge, this is the first reported case of pulmonary co-infection with MTB and SAG in an immunocompetent pediatric patient. Given the important implications in terms of clinical management and treatment outcome, both common (e.g. S.pneumoniae) and unusual pathogens (such as SAG) should be considered as potential co-infecting agents, particularly in high TB burden settings.

## Data Availability

All data generated or analyzed during this study are included in this published article. Besides, any additional data/files may be obtained from the corresponding author.

## Abbreviations

(ANC): Absolute neutrophil count
(CBC): Cell blood count
(CGD): Chronic Granulomatous Disease
(CRP): C-Reactive Protein
(CXR): Chest radiography
(E): Ethambutol
(ECG): Electrocardiogram
(INH): Isoniazid
(MRSA): Methicillin-resistant *Staphylococcus aureus*
(MTB): *Mycobacterium tuberculosis*
(PZA): Pyrazinamide
(RBC): Red blood cells
(RIF): Rifampicin
(SAG): *Streptococcus anginosus* group
(SCID): Severe Combined Immunodeficiency
(TB): Tuberculosis
